# Notch appearance as a novel radiological predictor of transient expansion and good outcome of expanding schwannoma after radiotherapy

**DOI:** 10.1007/s12672-024-00936-y

**Published:** 2024-03-19

**Authors:** Masahiro Yamazaki, Shigeyuki Takamatsu, Yuta Iwata, Takayuki Sakurai, Masashi Taka, Satoshi Kobayashi, Toshifumi Gabata, Eiichi Mizuno

**Affiliations:** 1 Department of Radiology, Kanazawa University School of Medical Science, Kanazawa City, Japan; 2 Toyama Prefectural Central Hospital, Toyama City, Japan; 3 Toyama CyberKnife Center, Toyama City, Japan

**Keywords:** Schwannomas, Notch appearance, Transient expanding tumor, Local control rate, Stereotactic radiotherapy

## Abstract

**Objectives:**

Schwannoma expansion after radiotherapy has not been well-studied despite the clinical importance of distinguishing transient increase from permanent expansion. Thus, this study aimed to identify the underlying mechanism and novel radiological predictors of schwannoma expansion after radiotherapy.

**Materials & methods:**

We retrospectively examined the therapeutic effects of radiotherapy on schwannomas and magnetic resonance images of 43 patients with vestibular schwannomas who underwent stereotactic radiotherapy or radiosurgery at our facility between June 1, 2012 and September 1, 2018. Based on the size change pattern, the treated tumors were classified into six groups, including transient-expansion and consistent-increase groups. The apparent diffusion coefficient (ADC) ratio and appearance of any notch were included as evaluation items based on our hypothesis that transient expansion is due to edema with increased extracellular free water. A log-rank test was performed to evaluate the relationship between the local control rate and radiological signs.

**Results:**

The mean overall 5-year local control rate was 90%, and the median follow-up period was 62 (24–87) months. Approximately 28% of the tumors showed transient expansion; all ADC ratios synchronized with size change, and 75% showed a new notch appearance. Approximately 9% of tumors showed consistent increase, with no notch on the outline. The log-rank test revealed a difference in the local control rate with or without notch appearance in expanding irradiated schwannomas. All tumors with notch appearance showed a significant regression 5 years after radiation.

**Conclusions:**

New notch appearance on the outline could indicate favorable long-term outcomes of expanding schwannomas post-treatment.

***** Clinical relevance statement***:**

Notch appearance can help differentiate a transient schwannoma from a real tumor expansion, and it is a novel predictor of better outcomes of expanding schwannomas after radiotherapy.

**Graphical Abstract:**

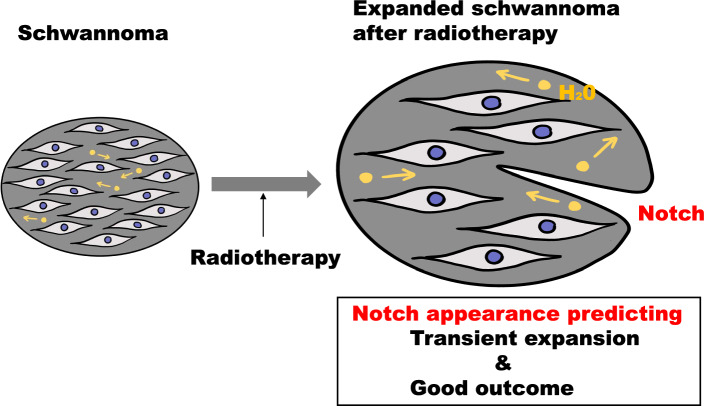

**Supplementary Information:**

The online version contains supplementary material available at 10.1007/s12672-024-00936-y.

## Introduction

Schwannomas are common nerve sheath tumors arising from Schwann cells [1–3]. The local mass effect causes various symptoms depending on the localization. For example, vestibular schwannomas (VS) can cause hearing loss, vertigo, and facial pain [2]. Compression of the brainstem or cerebellum by a schwannoma can be life-threatening and present as vomiting and coma [3, 4]. Stereotactic radiotherapy is an established treatment method for VSs because of its favorable effects and few adverse events [5–7]. Transient expansion, a temporal increase in tumor size, may occur after radiation therapy [8, 9]. Salvage surgery or e-irradiation is required if the growth continues. However, transient expansion is sometimes difficult to distinguish from a permanent increase owing to treatment resistance, and the surgery is complicated, with perioperative death occurring in some cases [10–12]. Therefore, it is clinically significant to understand the characteristics of this transient increase. Previous studies have suggested that pseudo-progression, a radiological change mimicking natural tumor progression in glioblastomas, may be a result of edema induced by damage to epithelial cells and local inflammation [13, 14]. However, few studies have reported the radiological signs of transient expansion of acoustic schwannomas after stereotactic radiotherapy, and the phenomenon has not been fully elucidated.

Thus, this study aimed to identify the underlying mechanism and novel radiological predictors. We hypothesized that the transient expansion of schwannomas is caused by edema with increased extracellular free water; the decrease in the volume of this extracellular free water triggers the onset of shrinkage. Under this hypothesis, the apparent diffusion coefficient (ADC) change would synchronize with the volume change, as the ADC value is correlated with the extracellular free water density and tumor cellularity [15, 16]. Therefore, we speculated that the tumor capsule tension would decrease around the peak of expansion. We tested this hypothesis via a retrospective review of magnetic resonance images. Furthermore, we evaluated the long-term efficacy of differentiating expanding schwannomas based on a new notch appearance on the outline.

## Materials and methods

### Study design and participants

This retrospective analysis was approved by the Ethics Review Committee of the study site (Toyama Nishi General Hospital Ethics Committee, Certificate number: 20–10). All study participants provided informed consent in written form. This study was performed in accordance with the relevant guidelines and regulations of this journal, including the principles of Declaration of Helsinki. This is a purely observatory study.

**Fig. 1:**
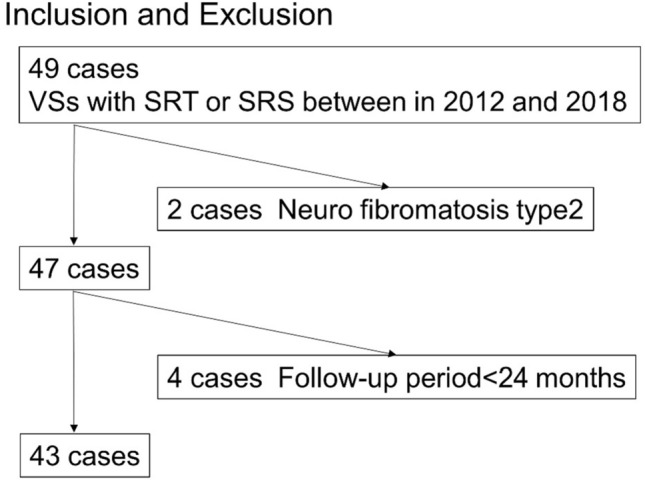
Study flow chart presenting patient eligibility. Data from 43 patients are analyzed. Two patients with type 2 neurofibroma are excluded. *VSs* vestibular schwannomas, *SRT* Stereotactic radiotherapy, *SRS* stereotactic radiosurgery

Among the 49 patients who underwent stereotactic radiotherapy and follow-up at XXX between June 1, 2012 and September 1, 2018 (for 80 months), data from 43 who were followed up for more than 2 years were subjected to image analysis. As the tumor control rate of type 2 neurofibromas is low [17, 18], the effects of radiotherapy on this type of tumor are distinct. Hence, two patients with type 2 neurofibromas were excluded from this study. The patient selection process is shown in Fig. 1.

### Magnetic resonance imaging equipment and imaging conditions

Magnetic resonance imaging (MRI) was performed using a 1.5-T MRI system (EXCELART VantageAtras, MRT-2003/S3; TOSHIBA,) with a head coil (Atlas SPEEDER MJAH-127A; Canon,). The same system was used for follow-up. Gadbutrol (Gadvist; Bayer) contrast material was injected, and gadolinium-enhanced T1-weighted MRI (Gd-T1WI) was performed 15 min after injecting the contrast medium. The imaging conditions were set as follows:

Gd-T1WI: repetition time (TR), 15–13 ms; time to echo (TE), 5.5 ms; flip angle, 25°; and slice thickness, 1 mm.

Three-dimensional (3D) steady-state free-precession T2-weighted MRI (3D-ssfpT2WI): TR, 12 ms; TE, 6 ms; flip angle, 75°; and slice thickness, 1 mm.

DWI: TR, 5800 ms; TE, 100 ms; flip angle, 90°; and slice thickness, 3 mm.

ADC: TR, 5800 ms; TE, 100 ms; flip angle, 90°; and slice thickness, 3 mm.

### Radiotherapy planning

Radiotherapy was performed using a treatment planning system (MultiPlan® version 4.6; Accuray Inc. Sunnyvale, CA, USA). The gross tumor volume was defined as the position and extent of the gross schwannoma; this was identified and contoured on computed tomography images with 1-mm-thick slices, which were fused with Gd-T1WI and 3D-ssfpT2WI images. CyberKnife® model G4, version 9.4 (Accuray, Inc.), was used for radiation. We aimed to ensure that 95% of the planning target volume (PTV) received 80% of the maximum dose (70–90% was acceptable). The PTV margin was set at 0‒1 mm, and we ensured that the minimum dose within the PTV did not fall below 10% of the prescribed dose. The radiological characteristics of the patients are summarized in Table 1.

**Table 1:** Radiotherapy planning characteristics

Prescription dose	14 Gy/1 Fr (n = 2)
	18 Gy/3 Fr (n = 29)
	25 Gy/5 Fr (n = 12)
	28 Gy/7 Fr (n = 1)
	Variables, median (range)
Number of fractions	3 (1–7)
Conformity Index	1.13 (1.02–1.25)
Homogeneity Index	1.25 (1.16–1.61)
Chair Heterogeneity Index	1.13 (1.08–1.30)

### Follow-up and local control rate

Follow-up was performed 3, 6, 12, 18, and 24 months after the end of the radiotherapy and yearly thereafter. The interval between follow-up observations was one of the most standardized features in this study (Online Resource 1). Before treatment, the tumors were stratified according to the extended Koos classification, and the 5-year local control rate of each group was studied [19]. Given that we considered that the prognosis of tumors displacing the brainstem differed according to whether they are large, the extended Koos classification, in which tumors compressing the brainstem were categorized as grade IV or V based on the volume, was used, with 6 cm^3^ as the benchmark [19].

### Imaging analysis

Two radiologists performed the imaging analysis. Volume changes were assessed using Gd-T1WI and 3D-T2WI, and the tumor volume was evaluated three-dimensionally by gross tumor delineation with study-planning software (MultiPlan, version 4.6). We defined “increase” and “decrease” as an increase or decrease in the tumor size volume by 13% or more, respectively, compared with the pretreatment values, as an inaccuracy of 13% has been reported during the manual delineation of volumes on high-resolution MR images [20, 21].

Treated tumors were classified into the following groups based on their size change patterns: Type A, increase before decrease; Type B, consistent decrease; Type C, no size change; Type D, consistent increase; Type E, decrease before increase; and Type F, increase after any decrease (recurrence). Transient expansion was defined as a significant decrease in size following a temporal increase in size (Type A). The interobserver agreement was evaluated by comparing the gross target volume delineated by the doctors with the benchmark determined by a trained medical physicist. All the stored MR images of Type A cases, which were taken at the pretreatment, peak of expansion, and last follow-up, were analyzed. To estimate the long-term efficacy of radiation, tumor size ratios (tumor volume 4 or 5 years after radiation/pretreatment) were defined and calculated. The ADC ratio was defined as the ratio of the mean signal in the region of interest to that in the contralateral thalamus. The region of interest was the entire tumor at the maximum cross-sectional level. Patients with any defect in the ADC image data were excluded.

We predicted that the tension of the tumor capsule would decrease around the transient expansion peak, and some imaging signs relevant to this decrease would appear. Therefore, the outlines and appearance of notches were included as evaluation items. For tumors with notch appearance, the time interval between the appearance of notches and significant shrinkage (13% volume decrease from the peak) was evaluated. Central nonenhancement (CNE) was defined as the appearance of low-signal areas in the tumor. For cystic tumors, the disappearance of enhancement at the septum was also regarded as CNE.

### Statistical analysis

The interobserver agreement was assessed using intraclass coefficient dice similarity coefficient (DSC), which is used to quantify the degree of tumor volume overlap. The following scale was set to evaluate the level of volume agreement (DSC ≥ 0.85, high agreement; ≥ 0.70 to < 0.85, moderate agreement; ≥ 0.50 to < 0.70, low agreement; and < 0.50, very low agreement) [22, 23]. The Friedman test and Dunn’s multiple comparisons test were performed to compare ADC ratios among pretreatment, tumor expansion peak during transient growth, and the last follow-up.

The Mann–Whitney test was performed to compare the tumor volume 4 or 5 years after irradiation to that at pretreatment. The log-rank test was performed to compare the differences in progression-free survival between patients with and without notch appearance. Individuals with missing data required for the analysis were excluded. All statistical analyses and graph making were performed using Graph Pad Prism 9, version 9.0.0 t or EZR [24], which is a modified version of R commander with added statistical functions. These are frequently used in biostatistics. For all analyses, the threshold for significance was set at a *P*-value of < 0.05.

## Results

### Patient characteristics

Data from a total of 43 patients who were followed up for more than 2 years were subjected to image analysis (Fig. 1). Table 2 shows the patient characteristics. Among them, 22 (51%) patients were male individuals, and the median age was 66 (interquartile range [IQR], 55–76) years. The median observation period was 62 (IQR: 28–64) months. Overall, the mean 5-year local control rate was 90% (95% confidence interval [95% CI], 70–97%). The 5-year local control rates for extended Koos grades I, II, III, IV, and V at our facility were 100%, 94%, 100%, 86%, and 80%, respectively (Online Resource 2). Among the patients with tumors that showed consistent growth, three patients underwent additional surgery or re-irradiation and were regarded to have local control failure. In a patient with grade V disease, hydrocephalus and brainstem edema appeared 11 months after irradiation; therefore, surgical management was performed, and this patient was also regarded to have local control failure.

**Table 2:** Patient and tumor characteristics

Variables	
Sex (male), n (%)	22 (51)
Median age (years) (range)	66 (31‒90)
Median KPS (range)	90 (70‒90)
Median Tumor volume	4.50 (0.20‒16.50)
Mean (cc) (range)	2.04 (0.20–16.6)
Extended Koos classification	
I	3
II	16
III	7
IV	13

The tumors were classified according to their size change patterns: Type A, 28% (12 of 43); Type B, 42% (18 of 43); Type C, 21% (9 of 43); Type D, 9% (4 of 43); Type E, 0% (0 of 43); and Type F, 0% (0 of 43). None of the Type A cases showed re-expansion, and the tumor expansion was transient. The interobserver agreement was assessed with the 36 tumor images and 72 delineations. The agreement was high, with a mean DSC of 0.95 (0.89–0.99, [95% CI]: 94%–96%). The ADC data were missing in five cases (Type A, one case; Type B, one case; Type C, two cases; and Type D, one case), and these cases were excluded from the analysis. Therefore, the ADC ratio was measured in 38 (88%) patients to test the hypothesis.

### Tumor diameter ratio 4–5 years after irradiation

In total, 9 Type A cases, 10 Type B cases, and 4 Type C cases were followed up for more than 4 years. The ratios of mean tumor sizes of Types A, B, and C in the fourth year after radiation to those in the pretreatment period were 0.51 (0.09–1.04, 95% CI: 0.26–0.75), 0.60 (0.29–0.80, 95% CI: 0.50–0.70), and 1.03 (1.13–0.96, 95% CI: 0.93–0.113), respectively. The extent of tumor size decrease in Type A was comparable with that in Type B.

A total of nine Type A cases, seven Type B cases, and two Type C cases were followed up for ≥ 5 years. The mean (range) tumor volume ratios of the Type A, B, and C cases in the fifth year were 0.45 (0.05–0.96, 95% CI: 0.21–0.70), 0.51 (0.23–0.71, 95% CI: 0.36–0.65), and 0.97 (0.95–0.99, 95% CI: 0.72–1.22%), respectively. The data are presented in Table 3.

**Table 3:** Tumor volume ratio after radiation, mean (CI)

	In 4 years	In 5 years
Type A	0.51(0.26–0.75)	0.45 (0.21–0.70)
Type B	0.60(0.50–0.70)	0.51 (0.36–0.65)
Type C	1.03(0.93–0.113)	0.97(0.72–1.22%)

### ADC ratio change during transient expansion

**Fig. 2:**
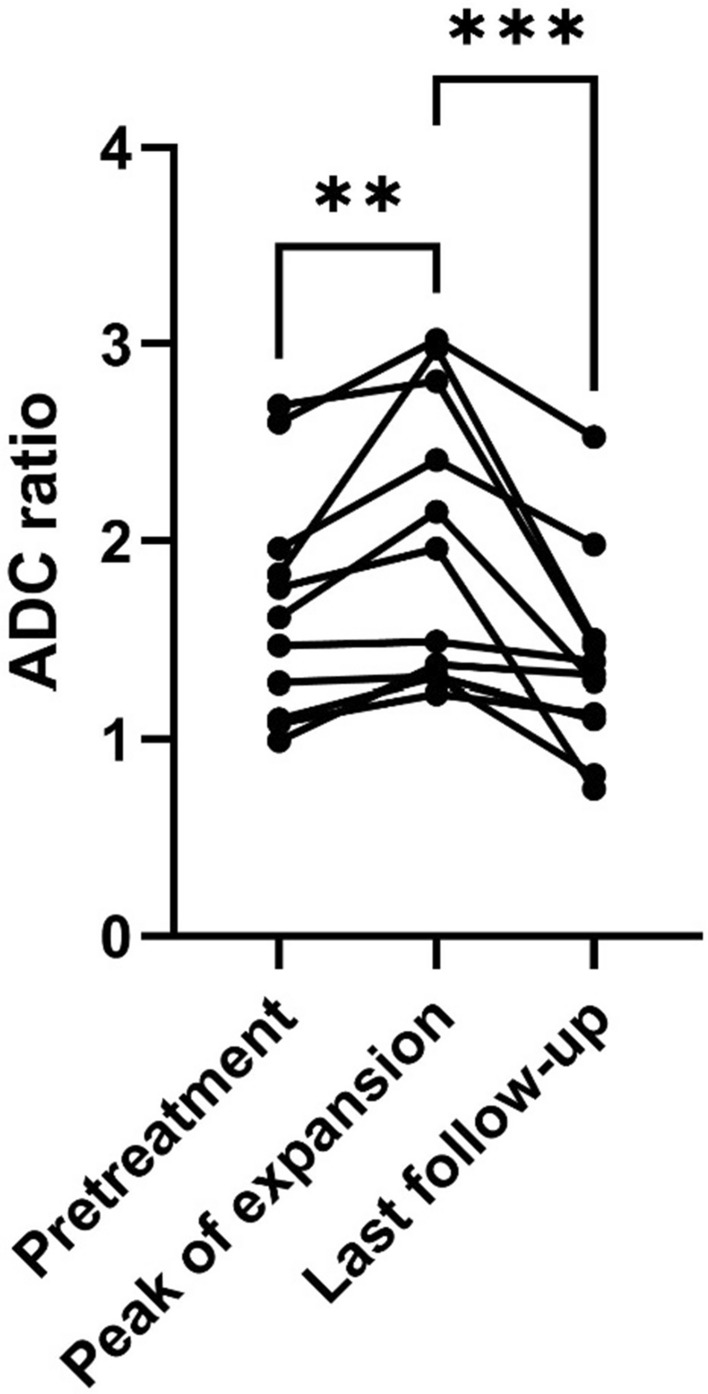
ADC ratio change in Type A (transient expansion) cases. A significant increase in tumor size is observed at the expansion peak, with a significant decrease observed at the last follow-up. **P = 0.008, ***P < 0.001; Dunn’s multiple comparisons test. *ADC* apparent diffusion coefficient

All Type A cases showed an increase in the ADC ratio during tumor growth, and the ADC ratio represented the maximum value at the peak of expansion (*P* = 0.008). In addition, the ADC ratio decreased as the tumor size reduced; consequently, a significant decrease was observed at the last time of follow-up (*P* < 0.001) (Fig. 2). The average tumor volume ratio between peak growth and pretreatment for Type A was 1.5 (95% CI: 1.3‒1.7), and the mean time to peak growth was 6.9 months (95% CI: 4.8‒9.0).

### Morphological changes during transient expansion

**Fig. 3:**
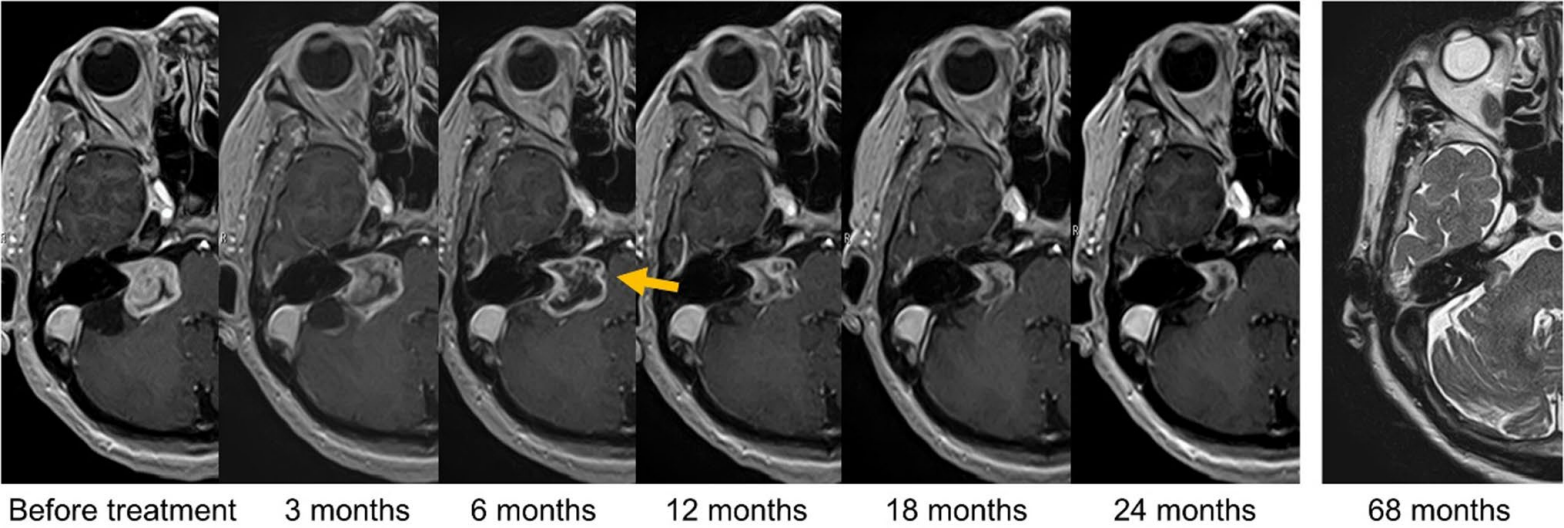
Typical example of a Type A tumor (transient expansion) in a 70-year-old woman. Gadolinium-enhanced T1-weighted imaging shows a notch appearance 6 months after stereotactic radiotherapy. The tumor relaxes and shrinks thereafter. No re-expansion is found within 68 months. A three-dimensional T2-weighted image shows no re-expansion at 68 months after radiotherapy (the last follow-up); no contrast medium was used as the patient did not provide consent

Nine Type A cases showed a new notch appearance, and 10 cases showed relaxation on the outline around the transient expansion peak (Fig. 3). After the emergence of these findings, all tumors continued to shrink and did not expand again. Four Type B cases showed a new notch appearance. No morphological signs were observed in the other types. In 67% of Type A cases with a notch appearance (6/9), notch formation occurred months before the significant decrease. In 70% of Type A cases with relaxation on outline (7/10), the tumors showed relaxing sign months before a significant decrease. The data are summarized in Table 4. CNE was observed in all cases that showed a change in tumor size. CNE was observed in all Type A, B, and D cases. Meanwhile, CNE was not observed in 5 of 9 (56%) Type C cases.

**Table 4:** Radiological signs by time interval before a significant decrease in patients with transient expanding neurinoma

	Time interval (months)					
	0	> 0 to ≤ 3	> 3to ≤ 6	> 6 to 12 ≤	> 12 to 18 ≤	> 18 to 24 ≤
Untensing on outline	3	0	2	3	2	0
Notch sign	3	0	1	2	2	1

### Long-term efficacy of radiation treatment for expanding schwannomas with a notch appearance

**Fig. 4:**
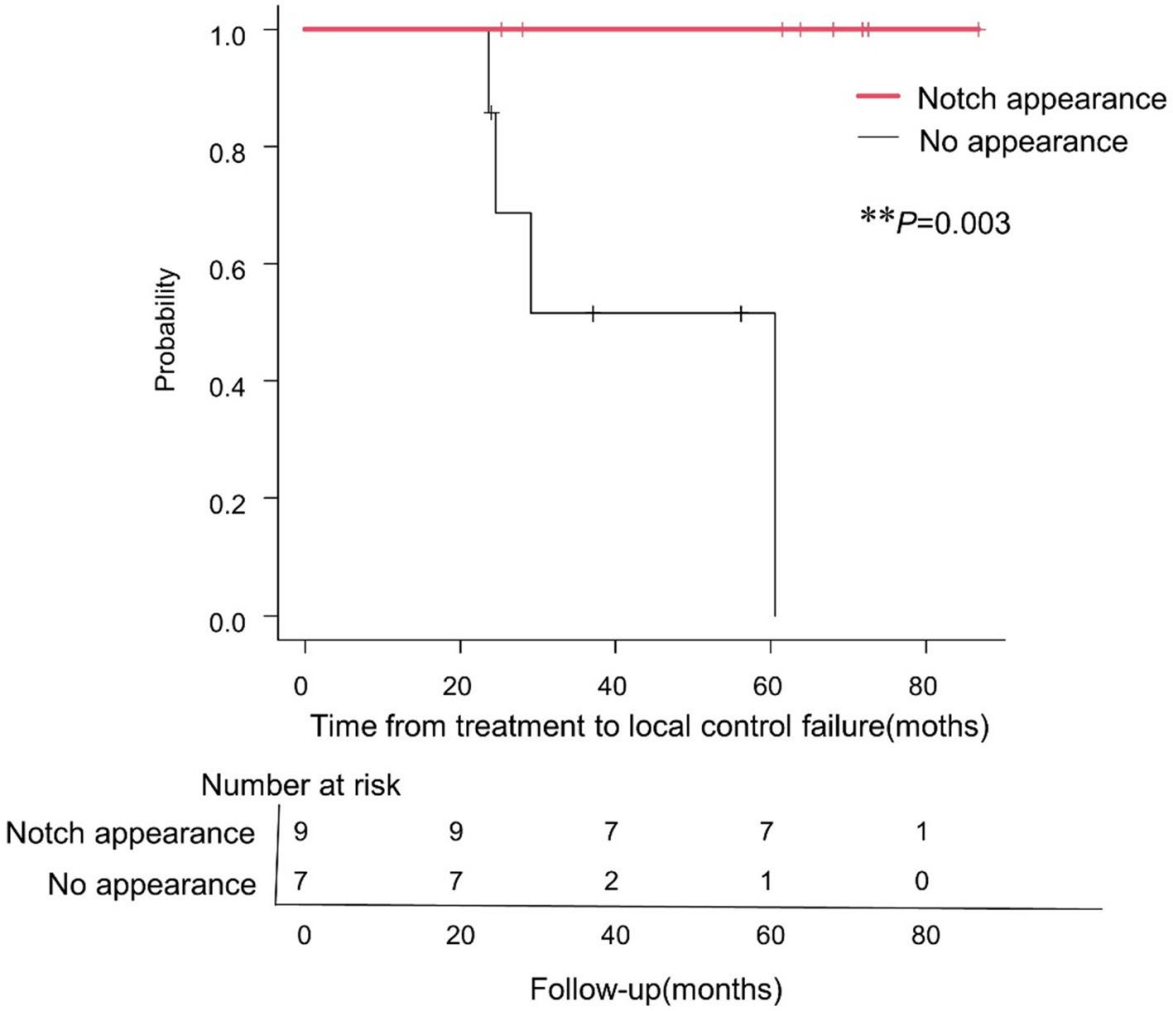
A Kaplan–Meier curve for overall survival in patients with expanding tumors with or without the notch appearance. The number at risk starts at 16 and decreases over time because of censoring or controlled failure. A significant difference was observed between the local control rate with and without notch appearance. **P = 0.003, log-rank test. Two patients with tumors having a notch appearance requested to stop watchful waiting by 24–40 months as they had no symptoms; moreover, the tumors did not re-expand. Six patients refused further follow-up at 60–89 months because the tumors did not show any re-expansion. These patients’ decisions were respected

The log-rank test revealed a difference between the local control rate of cases of irradiated expanding schwannomas with and without notch appearance (*P* = 0.003) (Fig. 4). All Type A tumors with notch appearance showed a significant decrease in the tumor volume ratio 5 years after radiation therapy (mean: 0.39 [range, 0.050–0.93], 95% CI: 0.16–0.61). Furthermore, no re-expansion was observed in the tumors with a notch appearance.

## Discussion

The local control rate of acoustic nerve tumors is 91%–99% [25–28]; when larger tumors are included, the rate is 82–98% [25]. In this study, the overall 5-year local control rate for vestibular schwannomas was 90%. These results are comparable with those reported by other institutes, and the radiological data are expected to reflect the general tendency of the treatment.

The aim of this study was to identify the underlying mechanism and novel radiological predictors. Our study suggests that the transient expansion of schwannomas can be a prognostic factor for good long-term outcomes. Some studies have reported that pseudo-progression in glioblastomas is more frequent with O6-methylguanine-DNA methyltransferase methylation, which tends to result in better outcomes [13, 29, 30]. However, to the best of our knowledge, no study has reported that transient expansion of schwannomas is associated with better tumor control. In our study, no Type A tumors showed re-expansion. In addition, all Type A tumors that were followed up showed a significant decrease in tumor size 4–5 years after radiotherapy. This data support our hypothesis that increased extracellular free water volume in the tumor causes transient expansion. During the process of transient expansion, the ADC of the tumors increases first and then decreases during tumor shrinkage. In our study, the ADC decreased after the expansion peak. At the peak, the tumors started to shrink, and the outline became rough; subsequently, they shrank sufficiently (Fig. 2). The findings imply that the extracellular free water volume temporarily increased after irradiation, and then the tumors started to shrink, and the tension of the tumor capsule decreased because of the absorption of the free water or leakage of the extracellular water.

Notch appearance and relaxation on the outline are novel prognostic indicators of transient expansion. No study has assessed radiological findings distinguishing real tumor growth from transient expansion. CNE has been reported as radiological changes in schwannomas after radiation treatment, and some reports have implied its relevance to subacute inflammation or vascular occlusion [6, 31]. However, the current study found that CNE could not rule out real tumor growth, as it appeared in all Type D tumors. In our study, notch appearance distinguished transient schwannomas from real tumor growth among expanding schwannomas (sensitivity, 75%; specificity, 100%) (Online Resource 3). In most cases, the sign appeared before significant shrinkage. All expansions with notch signs were transient, and tumors with transient expansion shrank sufficiently without re-expansion. Notch appearance and relaxation, denying actual tumor growth, will bevaluable radiological findings that may help in clinical decision making.

Gene expression profiling or histological analysis was not included in our study. Further studies are required to elucidate the molecular mechanism of transient expansion and its prognostic impacts.

## Conclusion

The appearance of any notch is a novel radiological predictor of good long-term prognosis of expanding vestibular schwannomas post-treatment. The radiological sign supports our hypothesis that transient expansion of schwannomas after radiation is caused by edema in the tumor.

### Supplementary Information

**Additional file 1.** Summary of previous and present studies’ follow-up timing and treatment outcomes, assessed with magnetic resonance imaging (MRI).

**Additional file 2.** Kaplan–Meier curves for tumor progression-free survival, according to the extended Koos classification. The software EZR is used to create the survival curves.

**Additional file 3.** Morphological signs in schwannomas showing first expansion (Type A or Type D).

## Data Availability

The data that support the findings of this study are available on request from the corresponding author. The data are not publicly available due to privacy reasons.

## Abbreviations

ADC: Apparent diffusion coefficient
TR: Repetition time
TE: Echo time
SRT: Stereotactic radiotherapy
SRS: Stereotactic radiosurgery
VS: Vestibular schwannomas
MRI: Magnetic resonance imaging
PTV: Planning target volume
Gd-T1WI: Gadolinium-enhanced T1-weighted image
CNE: Central nonenhancement
DSC: Dice similarity coefficient
IQR: Interquartile range
CI: Confidence interval
