# Supplementary material for: Notch appearance as a novel radiological predictor of transient expansion and good outcome of expanding schwannoma after radiotherapy
Source: Discov Oncol. 2024 Mar 19;15:79. doi: 10.1007/s12672-024-00936-y (PMC10951174; doi:10.1007/s12672-024-00936-y)
Supplement: Supplementary file 1 — Additional file 1. Summary of previous and present studies’ follow-up timing and treatment outcomes, assessed with magnetic resonance imaging (MRI). [file 12672_2024_936_MOESM1_ESM.pdf]

Name, year	Volume, median (cc)	Marginal dose	Follow-up MRI after radiosurgery	Follow up period	Hearing preservation rates (%)	Overall tumour control (%)
**Pollock et al, 2006**	1.5	Mean, 12.2	6, 12, 24, and 48 months then biannually	12–62 (mean, 42)	63	96
**Chopra et al, 2007**	1.3 (range, 0.08–37.5), previously untreated	12–13 (median, 13)	every 6 months for the first 2 years, then annually	median, 68 maximum, 143	57	99 at 10 years
**Rick et al 2011**	6.1–17.7 (mean, 8.8) << 4	10.3–13 (mean, 11.6)	biannually for the first 2 years and annually thereafter	12–72 (median, 30)	58	88
**Lipski et al, 2015**	1.3 (range, 0.2–6.1)	11–12 (mean, 11.5)	every 6 months during 2 years after treatment and then annually	24–84 (median, 48)	77	99 at 7 years
**Our study**	2.38 (range, 0.20–16.56)	11-20	3, 6, 12, 18, 24 months then at least once a year	14–87 (median, 50)	88	90

Online Resource 1
Summary of previous and present studies' follow-up timing and treatment outcomes, assessed with magnetic resonance imaging (MRI)
Discover Oncology, "Notch appearance as a novel radiological predictor of transient expansion and good outcome of expanding schwannoma after radiotherapy"
Masahiro Yamazaki, abearinthewoods_0419@yahoo.co.jp, Department of Radiology, Kanazawa University School of Medical Science, Kanazawa city, Japan
