# Supplementary material for: Notch appearance as a novel radiological predictor of transient expansion and good outcome of expanding schwannoma after radiotherapy
Source: Discov Oncol. 2024 Mar 19;15:79. doi: 10.1007/s12672-024-00936-y (PMC10951174; doi:10.1007/s12672-024-00936-y)
Supplement: Supplementary file 2 — Additional file 2. Kaplan–Meier curves for tumor progression-free survival, according to the extended Koos classification. The software EZR is used to create the survival curves. [file 12672_2024_936_MOESM2_ESM.pdf]

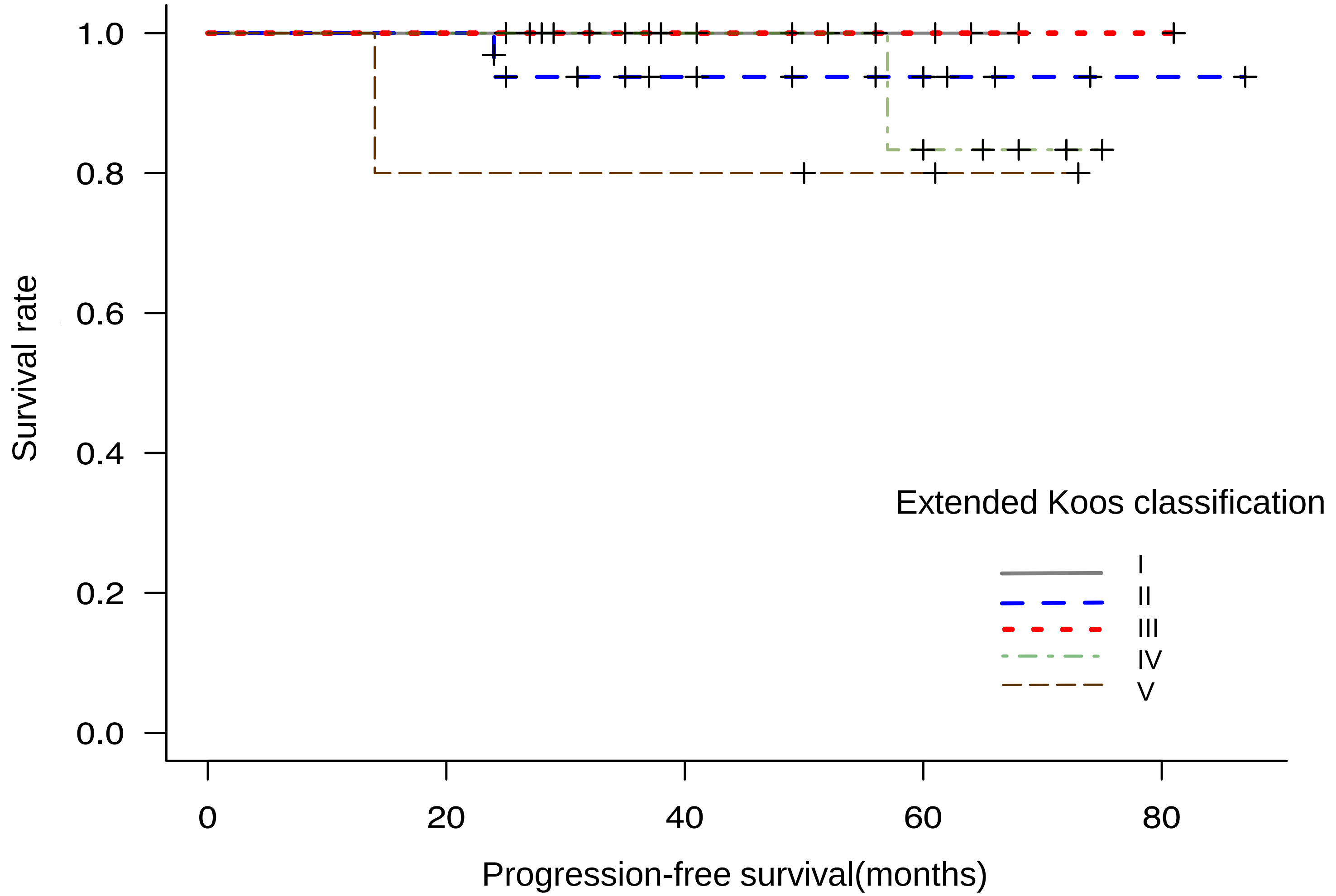

Online Resource 2
Kaplan–Meier curves for tumor progression-free survival, according to the extended Koos classification. The software EZR is used to create the survival curves.
Discover Oncology, “Notch appearance as a novel radiological predictor of transient expansion and good outcome of expanding schwannoma after radiotherapy” Masahiro Yamazaki, abearinthewoods_0419@yahoo.co.jp, Department of Radiology, Kanazawa University School of Medical Science, Kanazawa city, Japan
