# Supplementary material for: Notch appearance as a novel radiological predictor of transient expansion and good outcome of expanding schwannoma after radiotherapy
Source: Discov Oncol. 2024 Mar 19;15:79. doi: 10.1007/s12672-024-00936-y (PMC10951174; doi:10.1007/s12672-024-00936-y)
Supplement: Supplementary file 3 — Additional file 3. Morphological signs in schwannomas showing first expansion (Type A or Type D). [file 12672_2024_936_MOESM3_ESM.pdf]

Morphological signs of Type A and Type D

	Type A	Type D
Sign(+)	n=9<br>**Notch**<br>Sensitivity: **75%**<br>Specificity: **100%**<br><br>n=10<br>**Untensed**<br>Sensitivity: **83%**<br>Specificity: **100%**	n=0
Sign(-)	n=2	n=4

Online Resource 3

Morphological signs in schwannomas showing first expansion (Type A or Type D). Sensitivity: 100 × Number of Type A patients with tumors having a notch appearance or untensing/total number of Type A cases. Specificity: 100 × Number of Type D patients with tumors having no notch appearance or untensing /Total number of Type D cases. Notch sign and untensing showed a sensitivity of 75% and 83%, respectively, and a specificity of 100%.

Discover Oncology, "Notch appearance as a novel radiological predictor of transient expansion and good outcome of expanding schwannoma after radiotherapy" Masahiro Yamazaki, abearinthewoods_0419@yahoo.co.jp, Department of Radiology, Kanazawa University School of Medical Science, Kanazawa city, Japan
